# Social and Emotional Wellbeing Screening for Aboriginal and Torres Strait Islanders within Primary Health Care: A Series of Missed Opportunities?

**DOI:** 10.3389/fpubh.2017.00159

**Published:** 2017-07-07

**Authors:** Erika Langham, Janya McCalman, Veronica Matthews, Roxanne Gwendalyn Bainbridge, Barbara Nattabi, Irina Kinchin, Ross Bailie

**Affiliations:** ^1^Centre for Indigenous Health Equity Research, Central Queensland University, Cairns, QLD, Australia; ^2^The Cairns Institute, James Cook University, Cairns, QLD, Australia; ^3^The University of Sydney, University Centre for Rural Health - North Coast, Lismore, NSW, Australia; ^4^Western Australian Centre for Rural Health, University of Western Australia, Geraldton, WA, Australia

**Keywords:** social emotional wellbeing, mental health, indigenous health, screening management, primary healthcare services, health guidelines, health policy

## Abstract

**Background:**

Social and emotional wellbeing (SEWB) is a critical determinant of health outcomes for Indigenous Australians. This study examined the extent to which primary healthcare services (PHSs) undertake SEWB screening and management of Aboriginal and Torres Strait Islander clients, and the variation in SEWB screening and management across Indigenous PHS.

**Methods:**

Cross-sectional analysis between 2012 and 2014 of 3,407 Indigenous client records from a non-representative sample of 100 PHSs in 4 Australian states/territory was undertaken to examine variation in the documentation of: (1) SEWB screening using identified measurement instruments, (2) concern regarding SEWB, (3) actions in response to concern, and (4) follow up actions. Binary logistic regression was used to determine the factors associated with screening.

**Results:**

The largest variation in SEWB screening occurred at the state/territory level. The mean rate of screening across the sample was 26.6%, ranging from 13.7 to 37.1%. Variation was also related to PHS characteristics. A mean prevalence of identified SEWB concern was 13% across the sample, ranging from 9 to 45.1%. For the clients where SEWB concern was noted, 25.4% had no referral or PHS action recorded. Subsequent internal PHS follow up after 1 month occurred in 54.7% of cases; and six-monthly follow up of referrals to external services occurred in 50.9% of cases.

**Conclusion:**

Our findings suggest that the lack of a clear model or set of guidelines on best practice for screening for SEWB in Indigenous health may contribute to the wide variation in SEWB service provision. The results tell a story of missed opportunities: 73.4% of clients were not screened and no further action was taken for 25.4% for whom an SEWB concern was identified. There was no follow up for just under half of those for whom action was taken. There is a need for the development of national best practice guidelines for SEWB screening and management, accompanied by dedicated SEWB funding, and training for health service providers as well as ongoing monitoring of adherence with the guidelines. Further research on barriers to screening and follow up actions is also warranted.

## Introduction

The need for screening and management of social and emotional wellbeing (SEWB) concerns is highlighted by evidence that Australian Aboriginal and Torres Strait Islander (hereafter respectfully referred to as Indigenous) adults are three times more likely than non-Indigenous adults to experience very high levels of psychological distress (1, 2). Indigenous Australians are also two to three times more likely to attend emergency departments and be hospitalized for mental health problems, experience substance misuse-related conditions, and have more contact with community health services than other Australian adults (3–5). Social and wellbeing concerns contribute to the high burden of chronic disease and poor health status among Indigenous Australians, while conversely, people with chronic disease have a greatly increased risk of developing depression and other psychological problems (6, 7).

The concept of SEWB originated from the holistic view of Aboriginal health (8). The root cause of SEWB and effects of its absence were explained by Pat Anderson, Chairperson of Australia’s national Indigenous health research institute, the Lowitja Institute:

*Those of us who have worked on the frontline of Aboriginal health for any length of time know that beneath the surface reality of Aboriginal people’s poor health outcomes sits a deeper truth. It is about the importance of social and emotional wellbeing, and how this flows from a sense of control over one’s own life. Where this is lacking, as it is in so many Aboriginal families and communities, there is instead indifference and despair and a descent into poor lifestyle choices and self-destructive behaviours*… (Dr. Pat Anderson (9) p.v).

Social and emotional wellbeing has been defined as “a multidimensional concept of health that includes mental health, but which also encompasses domains of health and wellbeing such as connection to land or ‘country,’ culture, spirituality, ancestry, family, and community” (10). When SEWB is lacking, the resultant risk behaviors and outcomes can include: stress/distress, anxiety, depression, problem gambling, high risk alcohol consumption, recreational drug use, violence, and/or suicide (10).

Multiple Aboriginal health strategies and reports have recommended that primary healthcare services (PHSs), including government, non-government, and Aboriginal community-controlled health services (ACCHS), embed and integrate SEWB screening, early identification, and management of SEWB concerns as part of their preventive health focus (11–13). However, for a range of historical and funding reasons, PHSs have functionally separated SEWB service delivery, with physical health remaining the predominant clinical focus (6, 14). These include the historical influence of patterns of thinking about mental and physical health, as well as difficulties non-Indigenous health providers have had in reconciling conceptualizations of SEWB and mental health (6, 14). Funding for SEWB has been provided separately to broader health services, and often in fragmented, short duration grants. Unsurprisingly, the limited evidence available suggests that the integration of SEWB within PHSs has yet to be achieved. For example, a recent analysis of 17 Indigenous PHSs that used the Audit for Best Practice in Chronic Disease (ABCD) mental health audit tool (2012–2014), found that the majority of PHSs were lacking in priority aspects: client records and health summaries, risk factors and brief interventions, treatments, hospitalization and discharge, investigations, follow-up of abnormal results, and PHS systems (15). The report recommended system-level action to improve performance. Additionally, findings of chronic illness and maternal health audits identified SEWB screening and support as priority evidence-practice gaps (16, 17).

Screening for SEWB has gained significant attention in Australia (18–20) since it enables early identification and management of clients’ SEWB concerns. Evidence suggests that SEWB improvement may require a focus on both reducing stressors (risks) and improving capacity to cope with these, as well as enhancing protective factors (21). However, the assessment and management of SEWB is a complex and challenging task, due in part to a paucity of evidence about what might be considered evidence-based SEWB practice (22). There is an absence of national guidelines for SEWB screening and management (23), a paucity of suitable assessment tools (24), and a small number of published service or program evaluations (22). Given the lack of evidence, PHSs have managed SEWB concerns among their patients through diverse strategies such as formal screening approaches routinely or through health checks or informal and opportunistic screening. Other programs have included those that prevent and care for mental health problems and substance misuse, provision of community and cultural expertise, family support programs, and advocacy for housing, welfare, and other services (25).

Arguably the best available guidelines for SEWB screening are provided by Haswell-Elkins et al. (23), who modified the mental health protocols of the Royal Australian and New Zealand College of Psychiatrists and linked them with engagement and empowerment pathways to create protocols for the delivery of SEWB and mental health services in Indigenous communities. The guidelines recommend a pathway of a simple screening process for initial assessment, and/or a more comprehensive risk assessment for those with identified issues, to form the basis for a decision support tool including a social and emotional care plan and protocols for treatment and referral pathways.

This study utilizes data from a large-scale continuous quality improvement (CQI) program to examine the extent to which Indigenous clients of PHS are screened for SEWB concerns and actions taken to address identified concerns, and to identify factors associated with variation in SEWB screening and management. Although not nationally endorsed, we present our findings based on the pathway of care suggested by Haswell-Elkins et al. (23) to determine whether care provided by PHSs closely aligns with this pathway. We framed and tested four questions:

1. Within a 2-year period of the audit, have PHSs screened the majority of their Indigenous clients for SEWB concerns?
2. Is there substantial variation of SEWB screening across PHSs?
3. Is identification of SEWB concern managed in 100% cases through referral to an external or internal service or both?
4. Are clients followed up within 1 month for internal PHSs actions, and 6 months for external services referrals?

## Materials and Methods

### Data Source and Measures

This study utilizes data from the Audit and Best Practice for Chronic Disease (ABCD) project, a national research-based CQI initiative focused on improving Indigenous primary health care including systems for preventative health (26). The preventive health audit tool is used to audit medical records of PHS attendees aged 15 years and older, who have been a resident within the community where the PHS operates for at least 6 of the last 12 months (determined by attendance records and local staff knowledge), are not pregnant or 6 weeks postpartum at the time of the audit, and do not have a diagnosis of diabetes, hypertension, coronary heart disease, chronic heart failure, rheumatic heart disease, or chronic kidney disease. The full data set included 17,283 client records from 2005 to 2014 including PHS characteristics. These were filtered to only include records for clients who identified as Indigenous, who had been seen in the last 24 months, and who were aged between 15 and 65 years old. The data set was also filtered to only include the most recent audit for each PHS between 2012 and 2014 since SEWB screening has been introduced. This is a cross-sectional study, comprising a total of 3,407 Indigenous client records from a non-representative sample of 100 PHSs in four Australian states/territory, South Australia, Western Australia, Queensland, and the Northern Territory. The sample was stratified by gender.

The full dataset from the ABCD preventive health audit tool includes a range of variables relating to a number of other health behaviors, screening, and health outcomes. Only demographic, SEWB and PHS characteristic measures are described below.

Demographic characteristics of the clients included age, gender, Indigenous cultural identity, and the date of last attendance at the PHS.

#### SEWB Screening

In the ABCD preventive health audit protocol from SEWB screening is deemed to have occurred where there was documentation of a client being screened using a standard SEWB tool within the last 24 months. This was recorded as a dichotomous (yes/no) response. Six standard SEWB screening tools were specified: the Kessler 5, Kessler 6 (27), Kessler 10 (27, 28), Patient Health Questionnaire 2 (29), Patient Health Questionnaire 9 (30, 31), and Edinburgh Postnatal Depression Screen (32). However, the protocol allowed for the use of other standard tools and captured the name of the tool utilized in these cases.

#### SEWB Concern

Where a standard SEWB screening tool was not used, a dichotomous response (yes/no) captured whether there had been any other documented concern through informal or formal discussion about SEWB in the last 24 months.

#### SEWB Concern Follow Up Actions

Three items captured any follow up actions when SEWB risk was detected. These included recording of: (1) the type of follow up actions implemented; (2) a subsequent review within 1 month for internal PHS actions; and (3) a report for external service referrals within 6 months.

#### PHS Characteristics

Primary healthcare service characteristics included their location, governance structure (ACCHS or government operated), service population, length of accreditation, and participation in the ABCD CQI program. Location included both state/territory, and their Australian Statistical Geographical Classification to determine remoteness.

Ethics approval was obtained from research ethics committees in each jurisdiction [Human Research Ethics Committee of the Northern Territory Department of Health and Menzies School of Health Research (HREC-EC00153); Central Australian Human Research Ethics Committee (HREC-12-53); New South Wales Greater Western Area Health Service Human Research Committee (HREC/11/GWAHS/23); Queensland Human Research Ethics Committee Darling Downs Health Services District (HREC/11/QTDD/47); South Australian Aboriginal Health Research Ethics Committee (04-10-319); Curtin University Human Research Ethics Committee (HR140/2008); Western Australian Country Health Services Research Ethics Committee (2011/27); Western Australia Aboriginal Health Information and Ethics Committee (111-8/05); University of Western Australia Human Research Ethics Committee (RA/4/1/5051)].

### Statistical Analysis

Statistical analysis was conducted using SPSS V24 (IBM Corp, Armonk, NY, USA). Summary statistics were used to describe the client and PHS characteristics and SEWB screening rates. To compare rates of SEWB screening across the four States/Territory, an initial omnibus χ^2^ test of independence was conducted, followed by a *post hoc* pairwise test of proportions to understand significant difference. Multi-level logistic regressions were used to determine the level of association between predictor variables (PHS and client characteristics) and SEWB screening. Screening was compared for each characteristic using an unadjusted odds ratio (OR). Binary logistic regression was conducted to determine whether these relationships remained once jurisdictional and PHS characteristics were controlled for.

Cases that were screened for SEWB (*n* = 910) were then described using summary statistics. A composite indicator was created to determine SEWB concern based on a reported at risk response against one of the nominated screening tools, another standard screening tool, or other recorded concern. The prevalence of SEWB concern was compared between the States/Territory using an omnibus χ^2^ test of independence. Logistic regressions were then conducted to determine the level of association between client characteristic predictor variables and SEWB concern. Follow up actions for those clients where there was concern for their SEWB (*n* = 94) were described using summary statistics. Significance level was set at *p* < 0.05.

## Results

From the 3,407 audited client records in the sample, 50.2% were male, and 78% identified as Aboriginal. Ninety-nine percentage of Torres Strait Islander clients and 95% of clients who identified as both Aboriginal and Torres Strait Islander were located in Queensland. Cultural identity was therefore excluded as a predictor variable within the analysis to avoid the conflation of State/Territory influences. Twenty percentage of clients were aged 15–19 years; more than 46% of clients were between 20 and 35 years old, and only 8.7% were aged between 50 and 65 years. The majority of clients (79.4%) had been seen by the PHS within 6 months of the audit date. Further details of the client characteristics by jurisdiction are detailed in Table 1.

**Table 1:** Characteristics of clients by jurisdiction January 2012–December 2014.

		**Northern Territory**	**Queensland**	**South and Western Australia**	**Total**
		
	**Number of client records**	1,702	1,340	365	3,407

**Client level**	**% of client records audited**

Age at last appointment	15–19 years	17.8	22.5	21.1	20.0
	20–24 years	22.1	16.4	12.6	18.8
	25–29 years	18.7	13.6	11.5	15.9
	30–34 years	12.9	10.1	10.1	11.5
	35–39 years	8.4	9.5	9.0	8.9
	40–44 years	8.5	9.5	7.9	8.8
	45–49 years	7.3	6.6	11.0	7.4
	50–54 years	3.7	4.9	5.8	4.4
	55–59 years	0.5	4.5	7.7	2.8
	60–64 years	0.2	2.5	3.3	1.5
Gender	Male	49.5	49.9	54.5	50.2
	Female	50.5	50.1	45.5	49.8
Indigenous status	Aboriginal	99.4	44.9	99.2	78.0
	Torres Strait Islander	0.4	48.1	0.5	19.1
	Both	0.2	7.0	0.3	2.9
Last attendance	<6 months	86.8	75.6	58.9	79.4
	≥6 months	13.2	24.4	41.1	20.6

*Totals in columns may vary from 100% due to rounding effects*.

The majority (92%) of the PHSs were located in either the Northern Territory (48%) or Queensland (44%). In these States/Territory, most PHSs were in very remote areas (87.5 and 86.4%, respectively). Half of all PHSs were servicing populations of <500 people, and 84% were government operated. One PHS in Queensland was missing accreditation data. Only 30% of all PHSs had five or more CQI cycles. A full breakdown of PHS characteristics by jurisdiction is outlined in Table 2.

**Table 2:** Characteristics of primary healthcare services (PHSs) by jurisdiction January 2012–December 2014.

		**Northern Territory**	**Queensland**	**South and Western Australia**	**Total**
		
**PHS level**	**% of PHSs audited**	48	44	8	100

AGSC location	Major city	–	4.5	50.0	6.0
	Inner regional	–	2.3	–	1.0
	Outer regional	2.1	–	25.0	3.0
	Remote	10.4	6.8	–	8.0
	Very remote	87.5	86.4	25.0	82.0
Governance	Community-controlled	22.9	2.3	50	16.0
	Government	77.1	97.7	50	84.0
Length of accreditation^a^	Never accredited	64.6	11.4	12.5	37.0
	Accredited some of time	12.5	45.5	25.0	28.0
	Accredited all of time	22.9	40.9	62.5	34.0
Service population	≤500	54.2	50.0	25	50.0
	501–999	16.7	20.5	37.5	20.0
	≥1,000	29.2	29.5	37.5	30.0
CQI experience	Baseline	20.8	11.4	37.5	18.0
	2 cycles	18.8	4.5	25	13.0
	3 cycles	8.3	34.1	25	21.0
	4 cycles	16.7	22.7	–	18.0
	≥5 cycles	35.4	27.3	12.5	30.0

*Totals in columns may vary from 100% due to rounding effects*.

*AGSC, Australian standard geographical classification; CQI, continuous quality improvement*.

*^a^Accreditation with Australian General Practice Accreditation Limited, Quality Improvement Council, or other. No accreditation data were available for one QLD PHS*.

Rates of SEWB screening varied between States/Territory, rates of screening by jurisdiction are detailed in Table 3. An omnibus χ^2^ test of independence was used to evaluate whether the State/Territory within which the PHS was located were related to SEWB screening. The differences in rates of screening between jurisdictions were statistically significant, χ^2^ (2, *n* = 3,407) = 210.77, *p* < 0.001, although the association between State/Territory and screening was small Φ = 0.25.

**Table 3:** Screening of clients for social and emotional wellbeing by jurisdiction January 2012–December 2014.

	**Northern Territory**	**Queensland**	**South and Western Australia**
	
**Number of client records**	1,702	1,340	335

**% of client record audited**
Screened	37.1	13.7	24.9
Not screened	62.9	86.3	75.1

Client characteristics had little association with SEWB screening (Table 4). Higher levels of screening were significantly (*p* < 0.05) associated with the 20–24 [OR 1.29 95% confidence interval (CI) 1.01–1.65] and 25–29-year age groups (OR 1.33 95%CI 1.03–1.71). Not attending the PHS regularly (within the last 6 months) was associated with a significantly lower SEWB screening (OR 0.31 95%CI 0.24–0.39).

**Table 4:** Unadjusted regression analyses—client and primary healthcare service (PHS) characteristics relationship to social and emotional wellbeing (SEWB) screening.

***n* = 3,407 client records**				
		
**Outcome is client has been screened for SEWB in last 24 months**				
**Predictors**		**Unadjusted Odds ratio**	**95% confidence interval**	***p*-values**

**Client characteristics**				
Age at last appointment	15–19 years	1.00	(reference)	
	20–24 years	1.29	(1.01–1.65)	0.04
	25–29 years	1.33	(1.03–1.71)	0.03
	30–34 years	1.02	(0.77–1.36)	0.88
	35–39 years	0.92	(0.67–1.26)	0.60
	40–44 years	1.09	(0.80–1.49)	0.58
	45–49 years	0.99	(0.71–1.39)	0.98
	50–54 years	0.90	(0.59–1.38)	0.63
	55–59 years	1.39	(0.87–2.21)	0.16
	60–64 years	0.86	(0.43–1.72)	0.67
Last attendance	<6 months	1.00	(reference)	
	≥6 months	0.31	(0.24–0.39)	0.00
**PHS characteristics**				
Location	Urban	1.00	(reference)	
	Regional	1.28	(0.82–2.00)	0.28
	Remote	1.27	(0.90–1.79)	0.18
Governance	Community-controlled	1.00	(reference)	
	Government operated	1.53	(1.21–1.92)	0.00
Service population	≤500	1.00	(reference)	
	>500–999	0.49	(0.40–0.61)	0.00
	≥1,000	0.38	(0.31–0.46)	0.00
Length of accreditation	Never accredited	1.00	(reference)	
	Accredited some of time	0.50	(0.41–0.60)	0.00
	Accredited all of time	0.28	(0.23–0.34)	0.00
Duration of participation in ABCD continuous quality improvement project at time of audit	Baseline	1.00	(reference)	
	2 cycles	2.72	(2.06–3.58)	0.00
	3 cycles	1.62	(1.26–2.09)	0.00
	4 cycles	2.30	(1.76–3.02)	0.00
	≥5 cycles	1.70	(1.34–2.17)	0.00
**State/Territory**				
	Northern Territory	1.00	(reference)	
	Queensland	0.27	(0.22–0.32)	0.00
	South and Western Australia	0.56	(0.44–0.73)	0.00

A number of PHS characteristics had a significant relationship with variation in SEWB screening for a number of characteristics (Table 4). Lower rates of SEWB screening were associated with larger service populations (OR 0.38 95%CI 0.31–0.46). From a PHS systems perspective, those PHSs with longer periods of accreditation were associated with lower rates of SEWB screening (OR 0.28 95%CI 0.23–0.34). However, longer participation in CQI (number of completed cycles) was associated with increased rates of SEWB screening (OR 1.70 95%CI 1.34–2.17). Government operated PHSs were associated with higher levels of SEWB screening (OR 1.53 95%CI 1.21–1.92). Jurisdictional differences were significant (*p* < 0.01).

A binary logistic regression model was utilized to test the relationship between SEWB screening, PHS characteristics, and client characteristics, whilst controlling for the influence of State/Territory (Table 5). When State/Territory influence was controlled for, a number of PHS characteristics remained significantly related to SEWB screening. Lower rates of screening within the sample were significantly related to those PHSs servicing populations >1,000 people (OR 0.36 95%CI 0.30–0.43), and those who had participated in fewer CQI cycles. When both State/Territory and PHS characteristics were controlled for, lower screening rates were only significantly associated with clients who had not attended the PHS in the last 6 months (OR 0.40 95%CI 0.31–0.52), whilst higher screening rates for the 55–59-year age group were significant (OR 1.98 95%CI 1.22–3.23) (Table 5).

**Table 5:** Binary logistic regression analyses—client and primary healthcare service (PHS) characteristics relationship to social and emotional wellbeing (SEWB) screening.

***n* = 3,407 client records**				
**Outcome is client has been screened for SEWB in last 24 months**				
**Predictors**		**Odds ratio**	**95% confidence interval**	***p*-values**

**Client characteristics**				
Age at last appointment	15–19 years	1.00	(reference)	
	20–24 years	1.07	(0.83–1.38)	0.61
	25–29 years	1.13	(0.86–1.47)	0.39
	30–34 years	0.89	(0.66–1.21)	0.46
	35–39 years	0.83	(0.59–1.16)	0.28
	40–44 years	1.02	(0.74–1.42)	0.91
	45–49 years	0.94	(0.66–1.34)	0.73
	50–54 years	0.82	(0.53–1.27)	0.37
	55–59 years	1.98	(1.22–3.23)	0.00
	60–64 years	1.10	(0.54–2.26)	0.80
Last attendance	<6 months		(reference)	
	≥6 months	0.40	(0.31–0.52)	0.00
**PHS characteristics**				
Governance	Community-controlled	1.00	(reference)	
	Government operated	1.56	(1.27–2.02)	0.00
Service population	≤500	1.00	(reference)	
	>500–999	0.52	(0.43–0.65)	0.00
	≥1,000	0.36	(0.30–0.43)	0.00
Length of accreditation	Never accredited	1.00	(reference)	
	Accredited some of time	0.70	(0.56–0.87)	0.01
	Accredited all of time	0.36	(0.29–0.44)	0.00
Duration of participation in ABCD continuous quality improvement project at time of audit	Baseline	1.00	(reference)	
	2 cycles	2.48	(1.87–3.29)	0.00
	3 cycles	2.46	(1.88–3.22)	0.00
	4 cycles	2.41	(1.83–3.17)	0.00
	≥5 cycles	1.62	(1.27–2.06)	0.00

When factors associated with SEWB concern for those clients who had been screened were examined, no significant relationship between client characteristics (gender, age, cultural identification) and SEWB concern was identified. Prevalence rates of concern within the sub-sample (*n* = 907) varied considerably between States/Territory. The prevalence rates for each State/Territory (Table 6) show that rates of identified concern were considerably higher in the South and Western Australia sub-sample (45.10% clients). The differences in rates of SEWB concern between the States/Territory were significant, χ^2^ (2, *n* = 907) = 92.21, *p* < 0.001, and the association between States/Territory and SEWB concern was a medium effect Φ = 0.32.

**Table 6:** Prevalence of social and emotional wellbeing (SEWB) concern by jurisdiction January 2012–December 2014.

		**Northern Territory**	**Queensland**	**South and Western Australia**	**Total**
		
	**Number of screened client records**	632	184	91	907

**% prevalence of SEWB concern**
SEWB concern	Concern	9.0	10.9	45.1	13.0
	No concern	91.0	89.1	54.9	87.0

For the clients where SEWB concern was noted (*n* = 118), 26.3% received only further actions internal to the PHS; 11% were only referred to external services for further action; and 37.3% received both internal actions and an external referral (Table 7). No further actions were recorded for 25.4% of those clients with SEWB concerns.

**Table 7:** Actions recorded following social and emotional wellbeing (SEWB) concern by jurisdiction January 2012–December 2014.

		**Northern Territory**	**Queensland**	**South and Western Australia**	**Total**
		
	**Number of client records with SEWB concern**	57	20	41	118

**% of client records with SEWB concern**
Count of actions recorded	Primary healthcare service (PHS) action only	33.3	45.0	7.3	26.3
	External referral only	12.3	10.0	9.8	11.0
	Both PHS and external referral	35.1	35.0	41.5	37.3
	No actions recorded	19.3	10.0	41.5	25.4

Follow up actions taken for SEWB concerns were deemed to have occurred when there was a record of subsequent review within 1 month of PHS actions being taken, and a record of a report received from external services within 6 months. Percentage of clients with review or report by jurisdiction is shown in Table 8. Records of PHS review by 1 month ranged from 40 to 87.5%, with an overall average across the sample of 54.7%. Records of external reports received ranged from 47.6 to 55.6% with an average across the sample of 50.9%.

**Table 8:** Record of subsequent review or report following social and emotional wellbeing (SEWB) concern actions by jurisdiction January 2012–December 2014.

	**Northern Territory**	**Queensland**	**South and Western Australia**	**Total**

**% of records indicating subsequent review or report**
Primary healthcare service review	48.7	87.5	40.0	54.7
External report	51.9	55.6	47.6	50.9

## Discussion

This study aimed to determine a baseline of SEWB screening and management practice within PHSs serving Indigenous communities. Specific objectives included determining: the rate of screening Indigenous clients for SEWB concerns within a 2-year period; the rates of concern documented within clients who were screened; the actions taken to manage SEWB concerns identified; the follow up of these actions; and factors which influenced variation in practice. The aim and objectives of the present study were consistent with Haswell-Elkins et al.’s (23) recommended protocol for the management of SEWB within Indigenous communities. Whilst these guidelines have not been adopted nationally, they provide the best available guidance for the SEWB screening and management of Indigenous clients.

The absence of an evidence based, nationally directed protocol for managing SEWB in Indigenous communities means that there is no consistent guidance as to what constitutes best practice SEWB screening and management. National guidelines are standard in other areas of health concern. This lack of guidance could explain the variation in SEWB screening and management identified between jurisdictions, which are influenced more directly by local protocols. Whilst Northern Territory and South and West Australian PHSs had higher rates of screening than Queensland PHSs, they also had higher rates of no action taken on identification of SEWB concern. In corollary, Queensland PHSs had lower rates of screening but higher follow up than other jurisdictions.

Overall, only 26.6% of clients across the sample had been screened for SEWB within the 2-year period preceding the audit. The low proportion of Indigenous clients screened may be the result of a number of factors at the client, PHS, and system levels. These could include client suspicion about screening because of the past misuse of assessment to perpetuate stereotypes and impose political processes of social and cultural control (33), a lack of resourcing for the PHSs to provide culturally competent assessment, or low levels of confidence among practitioners in using existing screening tools for Indigenous clients (33).

A number of Indigenous developed/adapted screening tools are available (24, 34, 35). But there is concern that screening should occur as just one part of a broader culturally competent assessment process entailing: (1) formal training in culturally appropriate assessment; (2) a comprehensive client interview to explain and determine the appropriate assessment processes and any required screening; (3) reflective documentation of the process; and (4) interpretation and reporting of results using cultural explanations and avoidance of labeling (36). The low screening rate within the sample, however, means that it is likely that SEWB concerns are being under-diagnosed and that the gateway to SEWB service provision is thus being missed.

Two further concerns are worth highlighting. The first is the emphasis within the screening tools on identifying deficits and risk factors. Whilst these are useful in identifying SEWB concern, the identification of strengths and protective factors are also integral to a comprehensive assessment (23). Identification of these factors would also support a social and emotional care plan that contributes to empowerment and builds on extant strengths of the individual or community. The second concern is the focus on the individual. The broader issue of where to focus resources for SEWB has been highlighted by Westerman (37) who considered the benefits of focusing on individuals or broader, societal targets. Hunter (38) explored the competing demand for resources between the causes of social problems and the treatment of the downstream impacts.

Of particular concern, there was no record of any action taken for 25.4% of those with SEWB concerns. We cannot say definitively if this meant that nothing was done, whether the client refused actions or whether the action taken was just not recorded. Patterns of referral to either internal PHS actions or external services also varied between jurisdictions. Visit characteristics of when SEWB screening occurred may provide a better understanding of differences, and the implications for the clients that had no further action taken. However, it highlights that screening alone is insufficient, and establishing policy or practice guidelines around SEWB must identify appropriate strategies for supporting the development of social and emotional care plans, including protocols for treatment, referral pathways, and follow up with PHS staff.

Follow up of PHS and external referral varied both across States/Territory and between the type of action taken. Internal PHS follow up was 54.7% across the sample, however, this ranged from 40% in South and Western Australia to 87.5% in Queensland. Six-monthly follow up of referrals to external services was more consistent; the mean follow up rate was 50.9% and range was only from 47.6% in South and Western Australia to 55% in Queensland. It should also be highlighted that the record of follow up is not a record of any health outcome; merely that a process action has occurred. The audit process does not provide linked data on post-intervention client SEWB so it is not possible to determine what has changed for the client since the screening or actions taken occurred.

Embedding screening protocols into health service delivery is challenging in terms of the identification or development of best practice guidelines, the training of staff to deliver socially and culturally appropriate screening, and the additional resources required to enable this in an already resource constrained system. Screening for SEWB, and the management of SEWB concerns is particularly sensitive given the need for it to occur within an appropriate therapeutic relationship. Haswell-Elkins et al.’s (23) guidelines recommend a pathway from an initial assessment utilizing culturally appropriate screening, through a more comprehensive assessment when SEWB concern is identified; to the development of a social and emotional care plan and protocols for treatment and referral pathways. This protocol offers a more culturally appropriate, consistent, and effective approach to SEWB screening and management than has been identified in this study. The adoption of a consistent and effective protocol, however, requires national direction; dedicated funding; and ongoing monitoring and evaluation to ensure improvements on the baseline established through this study.

### Limitations

Some important limitations to the interpretation of these data are worth highlighting. The data set is not a representative sample of Indigenous PHSs across the whole of Australia or even those within each of the States/Territory. It only captures those services that have been part of the ABCD research program. The findings should not be generalized to all Indigenous PHSs nationally, or even within each jurisdiction. Additionally, the data set only includes audits up to 2014, and therefore does not reflect any improvements in SEWB screening and management of SEWB concern that have been achieved since that time. Also lack of access to health center data on funding models precluded us from analyzing the impact of funding on variation in service provision.

Between the levels of the client characteristics and the PHS characteristics, are what could be referred to as the visit characteristics. These would include the reason for the client’s visit to the PHS, and the type of health professional they first saw when SEWB screening occurred. Within the current data set, only details of the client’s last visit is included, however, it is not possible to determine if this is the visit at which SEWB screening occurred. Additional data on these visit characteristics would provide a better understanding of differences in screening practices, and offer utility in being able to identify implications for establishing best practice guidelines. For example, if more screening was being carried out by Aboriginal and Torres Strait Islander Health Workers than Nurses or GPs, then that would provide a clear direction that investing in more health worker positions could assist in improving screening rates. Similarly, if screening was occurring more during well person checks than acute care visits then again that would suggest strategies to increase the use of well person checks such as more health workers, or client education.

## Conclusion

Social and emotional wellbeing screening is a complex and sensitive process that should occur within a comprehensive and culturally sensitive client interview. The findings of the present study suggest that there is a lack of clarity regarding best practice pathways for SEWB screening and management, and there are a number of opportunities to improve key aspects. The results tell a story of missed opportunities: 73.4% of clients were not screened; no further action was taken for 25.4% of those screened for whom an SEWB concern was identified; and there was no follow up for nearly half of those for whom actions were taken. The results suggest a clear need for national best practice guidelines for SEWB screening and management, accompanied by dedicated SEWB funding, and training for health service providers.

Future research is needed to link SEWB screening protocols to health outcomes. At present, the data track a health service process outcome of whether client follow up has been actioned. However, there is no visibility of the outcome of the actions taken in terms of the client’s wellbeing, such as changes to levels of psychosocial distress or depression. A clearer understanding of the link between the health service processes and health outcomes is important in guiding clinical practice and being able to assess the cost-effectiveness of investing in service initiatives.

## Ethics Statement

Ethics approval was obtained from research ethics committees in each jurisdiction [Human Research Ethics Committee of the Northern Territory Department of Health and Menzies School of Health Research (HREC-EC00153); Central Australian Human Research Ethics Committee (HREC-12-53); New South Wales Greater Western Area Health Service Human Research Committee (HREC/11/GWAHS/23); Queensland Human Research Ethics Committee Darling Downs Health Services District (HREC/11/QTDD/47); South Australian Aboriginal Health Research Ethics Committee (04-10-319); Curtin University Human Research Ethics Committee (HR140/2008); Western Australian Country Health Services Research Ethics Committee (2011/27); Western Australia Aboriginal Health Information and Ethics Committee (111-8/05); University of Western Australia Human Research Ethics Committee (RA/4/1/5051)].

## Author Contributions

EL contributed to project design, analyzed the data, contributed to interpreting the data, drafting and critically revising the manuscript. JM contributed to project design, interpreting the data, drafting and critically revising the manuscript. VM critically reviewed and commented on the project design and critically revised the manuscript. RB contributed to interpreting the data and critically revising the manuscript. BN commented on the project design and critically revised the manuscript. IK commented on the project design and critically revised the manuscript. RB critically reviewed and commented on the project design and on the manuscript. All authors read and approved the final manuscript.

## Conflict of Interest Statement

The authors declare that the research was conducted in the absence of any commercial or financial relationships that could be construed as a potential conflict of interest.
